# Generalized estimation of the ventilatory distribution from the multiple-breath washout: a bench evaluation study

**DOI:** 10.1186/s12938-018-0442-3

**Published:** 2018-01-15

**Authors:** Gabriel Casulari Motta-Ribeiro, Frederico Caetano Jandre, Hermann Wrigge, Antonio Giannella-Neto

**Affiliations:** 1 Pulmonary Engineering Laboratory, Biomedical Engineering Programme, COPPE, Universidade Federal do Rio de Janeiro, Rio de Janeiro, Brazil; 2 Department of Anesthesiology and Intensive Care Medicine, University of Leipzig, Leipzig, Germany

**Keywords:** Pulmonary function tests, Ventilatory distributions, Multiple-breath washout, End-expiratory lung volume, Functional residual capacity, Dead space, Nitrogen, Ventilation to volume, Tikhonov regularization, Common dead space

## Abstract

**Background:**

The multiple-breath washout (MBW) is able to provide information about the distribution of ventilation-to-volume (v/V) ratios in the lungs. However, the classical, all-parallel model may return skewed results due to the mixing effect of a common dead space. The aim of this work is to examine whether a novel mathematical model and algorithm is able to estimate v/V of a physical model, and to compare its results with those of the classical model. The novel model takes into account a dead space in series with the parallel ventilated compartments, allows for variable tidal volume (V_T_) and end-expiratory lung volume (EELV), and does not require a ideal step change of the inert gas concentration.

**Methods:**

Two physical models with preset v/V units and a common series dead space (v_d_) were built and mechanically ventilated. The models underwent MBW with N_2_ as inert gas, throughout which flow and N_2_ concentration signals were acquired. Distribution of v/V was estimated—via nonnegative least squares, with Tikhonov regularization—with the classical, all-parallel model (with and without correction for non-ideal inspiratory N_2_ step) and with the new, generalized model including breath-by-breath v_d_ estimates given by the Fowler method (with and without constrained V_T_ and EELV).

**Results:**

The v/V distributions estimated with constrained EELV and V_T_ by the generalized model were practically coincident with the actual v/V distribution for both physical models. The v/V distributions calculated with the classical model were shifted leftwards and broader as compared to the reference.

**Conclusions:**

The proposed model and algorithm provided better estimates of v/V than the classical model, particularly with constrained V_T_ and EELV.

**Electronic supplementary material:**

The online version of this article (10.1186/s12938-018-0442-3) contains supplementary material, which is available to authorized users.

## Background

The estimation of the pulmonary ventilation-to-volume (v/V) distribution may provide clinically useful information on intrapulmonary gas-mixing but is an underused byproduct of the end-expiratory lung volume (EELV) measurements during mechanical ventilation. The v/V can be calculated with the multiple-breath washout (MBW) test, especially using N_2_ as the inert and low solubility gas (MBN_2_W). The classical method [1–3] models the lungs as a set of all-parallel units, including a dead space, whose contributions to the total lung ventilation are the unknowns. This approach has some limitations. For instance, it disregards the effects of the series dead space (v_d_), whose volume may be estimated via the Fowler’s method [4] throughout the washout; not only the EELV but also the tidal volume (V_T_) must remain constant during the MBN_2_W; the inspired fraction of tracer gas should decrease instantaneously to zero. Recently, we [5] proposed a generalized multicompartmental model for MBN_2_W that includes a series dead space and copes with a non-ideal step change in gas concentration, variable V_T_ during the maneuver, and changes in EELV, as long as no compartment is completely emptied. Computational simulations showed that this model, together with an algorithm to estimate its parameters from measurements taken at the airway opening during MBN_2_W, usually retrieved more correct estimations of the v/V distribution than previous proposals [5]. Furthermore, the alternative to impose a priori constraints determined along the MBN_2_W limits the set of the v/V parameters estimates. However, since this same novel model drove the simulated MBN_2_W, the results could have favored the algorithm in some form. It is arguable, hence, that bench tests with well-known physical models would allow for a better, less biased assessment of the effects of modelling the series dead space in the estimates of v/V distributions.

The present work intends to compare the v/V distributions estimated by both the classical and generalized approaches employing experimental data obtained from physical models, under the conditions (constant V_T_ and EELV) required by the assumptions of the classical model. Similar estimation procedures were used for both models, employing non-negative least squares and Tikhonov regularization plus a weighting matrix. The generalized approach adds a constrained least squares solver with imposed EELV, V_T_ and v_d_. The results previously obtained by us [5], with numerically simulated experimental noise, directed the choice of the weighting matrix.

## Methods

### Mathematical model of the MBN_2_W

The generalized mathematical model of the MBN_2_W is as follows. The respiratory system comprises N parallel compartments, all connected through a single duct whereby the gases are exchanged with the ambient air. Each compartment J, whose volume is Vol_J_, is an ideal mixer characterized by the fraction *γ* of V_T_ that enters and leaves it at each cycle, and its specific ventilation (*S*(*J*) = *γV*_*T*_/*Vol*_*J*_), the sum of all compartmental volumes being equal to EELV-v_d_. A series dead space is incorporated, considering that a compartment inspires a mixture of fresh gas from the inspiratory circuit and the content of the common duct. This also allows the model to be driven by a non-ideal step in inspiratory concentration of the tracer gas. Variable V_T_ is admitted by defining S(J) with respect to a reference V_T_, and variable EELV is achieved by tracking the differences between inspired and expired volumes, returning the distribution corresponding to EELV at the onset of maneuver [5].

In the experimental setup, where V_T_ and EELV were constant, the end-tidal N_2_ concentration ($F_{{N_{2} }}^{et}$) at the k-th cycle is modeled by

1 $$F_{{N_{2} }}^{et} (k) = \mathop \sum \limits_{J = 1}^{N} \gamma (J) F_{{N_{2} }}^{A} (J,k),$$

with the compartmental concentrations given by

2 $$F_{{N_{2} }}^{A} (J,k) = \frac{{\left( {F_{{N_{2} }}^{et} (k - 1)\alpha + F_{{N_{2} }}^{I} (k)(1 - \alpha )} \right)S(J) + F_{{N_{2} }}^{A} (J,k - 1)}}{1 + S(J)}$$

where $\alpha$ is the dead space to tidal volume ratio (v_d_/V_T_).

The classical approach to model multiple compartment MBN_2_W considers an ideal step change of the inspired tracer gas at the onset of washout with the dead space as an additional parallel compartment. Under these assumptions, Eq. 2 simplifies to

3 $$F_{{N_{2} }}^{A} (J,k) = \frac{{F_{{N_{2} }}^{A} (J,k - 1)}}{1 + S(J)}$$

and the combined compartmental concentrations are fitted to the measured mean expiratory N_2_, by adjusting the respective weights. For a single compartment with a series dead space, it can be demonstrated, by using Eqs. 2 and 3, that this classical parallel model estimates a compartment with ventilation ($1 - \alpha$) shifted leftwards (lower specific ventilation) from the real compartment. The estimated specific ventilation (S′) depends on the actual specific ventilation (*S*′ = (1 − *α*) · *S*/(*αS* + 1)), causing larger differences for faster compartments. Accordingly, the estimated compartmental volume is equal to EELV.

In case of a non-ideal step at onset of washout, a further shift depending on the ratio of inspired to expired concentrations occurs. To distinguish partially between this effect of a non-ideal step and the presence of a series dead space, an alternative classical model was tested. This is modeled by Eq. 2 with *α* = 0.

### Experimental setup

To test the effect of a series dead space in the washout maneuver under controlled conditions, two physical models were assembled: one with four compartments of equal *γ* and different Vol_J_ (4C); and one with a single compartment (1C). The 4C allowed to examine the recovery of location, and the spread/breadth of the distribution, while with 1C the classical model distribution shift could be analytically predicted. Both models were ventilated by an Evita XL (Draeger Medical, Lübeck, Germany) and N_2_, O_2_ and CO_2_ concentrations were measured by a fast mass spectrometer (AMIS 2000, Innovision, Glamsbjerg, Denmark). Pressure and flow signals were acquired directly from the ventilator and with a proximal pneumotachograph plus a pressure transducer. In order to synchronize the signals of gas concentration and flow, an uncalibrated flow signal was recorded from a pneumotachograph connected to the mass spectrometer, and the mainstream capnometer from the ventilator was placed close to the gas sampling port. All data were recorded simultaneously with a program written in LabView (National Instruments, Austin, USA).

The ventilated compartments were 1-L anesthetic bags (VBM Medizintechnik GmbH, Sulz am Neckar, Germany) with end-expiratory volume maintained by application of a positive end-expiratory pressure (PEEP). A super-syringe inflation determined that at PEEP of 10 cmH_2_O the volume of the bag was 1 L. CO_2_ production was simulated by a constant low flow of this gas into the compartment with the smallest v/V ratio. CO_2_ flow was titrated to achieve end-tidal concentration between 0.5 and 1% to reduce effects in expired volume.

**Fig. 1:**
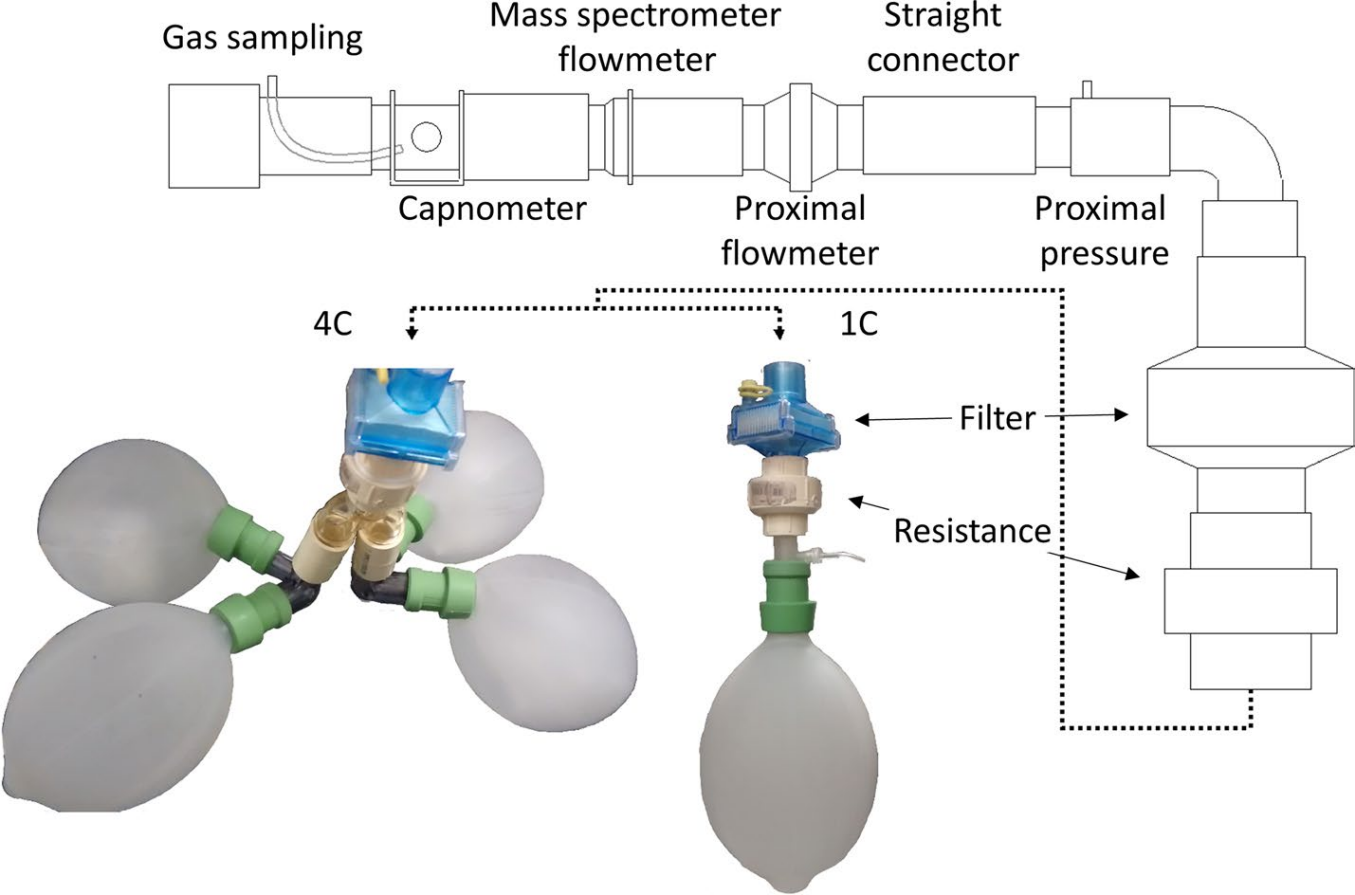
Representation of the experimental setup. The physical models are shown as photos with the anesthetic balloons at end-expiration. The components of the series dead space are represented by schematic drawings. The Y-piece of the ventilatory circuit was connected directly to the gas sampling piece. Note that the gas is sampled close to the capnometer chamber to avoid inspiratory/expiratory delay changes

The series dead space comprised an anatomical and an instrumental dead space. The anatomical dead space was represented by a resistive piece and standard connectors used in mechanical ventilation, such as 22-to-15 mm reductions and Y-pieces. The instrumental dead space was the connector for sidestream gas sampling and the pneumotachograph of the mass spectrometer, the mainstream capnometer of the ventilator, the proximal pneumotachograph and pressure outlet, a 90° connector to the resistance, and an HME filter (BB25, Pall Medical, Port Washington, USA) (Fig. 1). The total dead space volume (v_d_), calculated from the geometry, were of 92 mL for 1C and of 152 mL for 4C.

Tidal volume and end-expiratory volume of compartments were selected to match, as nearly as possible, specific compartments from a logarithmic distribution of *N* = 50 ventilation-to-volume ratios ranging from 0.01 to 100. The 1C model was ventilated with V_T_ = 250 mL, representing a compartment with S = 0.25. The respiratory frequency was of 15 breaths/min. A total of 5 washouts were performed with a N_2_ step change from 50% to zero. The 4C model compartments had 1.00, 0.83, 0.69 and 0.57 L and were ventilated with V_T_ = 560 mL, or 140 mL per compartment (S = 0.14, 0.83, 0.69 and 0.57, respectively). Where applicable, the compartmental end-expiratory volumes available for gas washout were reduced by inserting closed, impermeable plastic containers (Profissimo Gefrierbeutel, Germany) into the anesthetic bags, filled with appropriate volumes of air. The respiratory frequency was of 12 breaths/min (7 tests), 10 breaths/min (3 tests) or 15 breaths/min (2 tests). A total of 12 washouts were performed. In 9 tests, the N_2_ step change was from 50% to zero and in 3 tests the step change was limited from 10% to zero. Experiments were performed in ATPD conditions, disabling the ventilator’s BTPS compensation.

### Signal processing

Before the data analysis, gas concentrations and flow were synchronized with a two-step procedure. First, flow curves from the ventilator and the mass spectrometer were aligned by maximizing their cross-correlation. Second, the delay from gas sampling was compensated breath-by-breath using the cross-correlation between the CO_2_ signals from the mass spectrometer and the ventilator mainstream sensor.

The synchronized signals were processed to estimate v_d_, EELV and the *γ* values of the compartments. The v_d_ was calculated from CO_2_ and volume curves using Fowler’s method [4]. The EELV was estimated from inspired and expired N_2_ volume during the washout (from onset until a N_2_ concentration ≤ 1/40th of initial value) [6]. Analogously, the distributions were estimated using the same number of cycles. The parameters of the multiple compartment model were estimated with nonnegative least squares and Tikhonov regularization with a fixed gain (4 × 10^−3^ for 1C and 3.3 × 10^−2^ for 4C) and a weighting matrix proportional to the compartmental washout ratio [2]. The generalized model was also estimated with a constrained least squares solver, imposing the sum of compartmental volume equal to the EELV-v_d_ and unitary total ventilation [5]. Overall resistance and elastance were calculated from pressure and flow signals to ensure similar mechanical behaviors of the compartments. Data were analyzed in MatLab (Mathworks, USA).

## Results

**Fig. 2:**
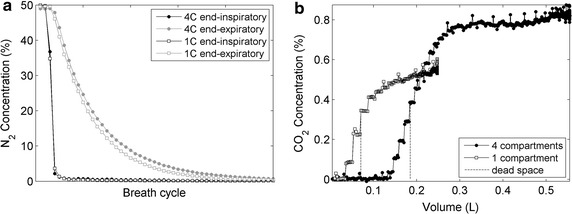
Examples of N_2_ washout and CO_2_ versus volume curves for the single (hollow square) and four compartment (filled square) physical models. **a** Inspiratory (black) and expiratory (gray) end-tidal N_2_ fractions during one washout maneuver of each model. **b** Expired CO_2_ versus volume, the dashed line represents the dead space volume as calculated by Fowler’s technique

The time profile of inspiratory N_2_ was not that of an ideal step and, as expected, the washout of 4C was slower than that of 1C (Fig. 2a). EELV was estimated, from the MBN_2_W inspired and expired N_2_ volumes, as 1.13 + 0.01 L for 1C and 3.24 ± 0.07 L for 4C. Typical expiratory capnogram curves were observed, despite the difference in magnitude (Fig. 2b). The estimated v_d_ were 73.8 ± 6.4 mL for 1C and 185.7 ± 4.5 mL for 4C (see the Additional file 1: Tables S1 and S2, for individual estimates of each experiment). The calculated overall resistance and elastance were R = 16.6 ± 0.3 cmH_2_O/L/s and E = 78.5 ± 1.2 cmH_2_O/L for 1C and R = 16.1 ± 0.6 cmH_2_O/L/s and E = 20.8 ± 0.3 cmH_2_O/L for 4C.

**Fig. 3:**
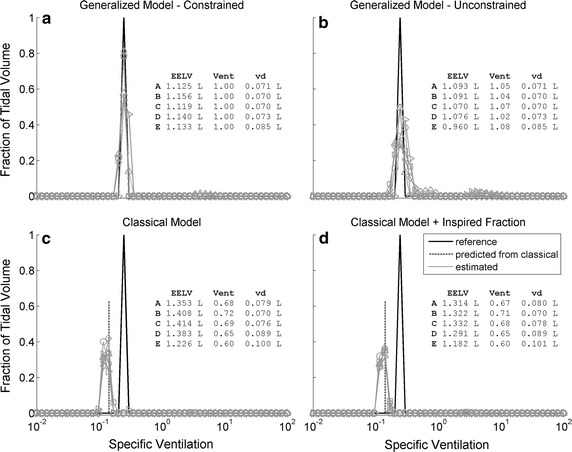
Distribution of specific ventilation estimated from the N_2_ washout of a single compartment physical model. Results from each of five (A to E) repetitions are represented in gray with different symbols. The reference distribution is shown in black. The vertical dashed line (panels **c** and **d**) represents the theoretical distribution predicted for the compartment estimated by the classical model (ideal step washout). EELV is the end-expiratory lung volume; vent is the sum of the fractional compartmental ventilations (∑γ); and v_d_ is the dead space volume estimated by: Fowler’s technique (for the generalized model, panels **a** and **b**) or the complement of total ventilation (for the classical model, panels **c** and **d**)

The distribution retrieved by the constrained generalized model for physical model 1C was located at the correct compartment, with a small contribution of an adjacent compartment, and corresponded to the smallest sum of squared errors between estimates and the real distribution of v/V (Fig. 3a and Table 1). In the case without constraints, the sum of compartmental volumes plus v_d_ underestimated EELV by 3%, and the total ventilation was overestimated by 5% (Fig. 3b). The classical model retrieved two or three compartments, located, however, leftwards from the actual compartment, as theoretically predicted (Fig. 3c). EELV was overestimated by 24%, and v_d_ (complement of total ventilation) was underestimated by 10%. The inclusion of the inspired N_2_ concentration partially corrected EELV estimations (mean error of 18%), but the distribution almost did not change (Fig. 3d). The estimated distribution of each test with each model is shown in the Additional file 1: Figures S1–S4.

**Table 1:** Sums of the squared errors between the estimated and true ventilation-to-volume ratio distributions

	Single compartment (1C)	Four compartments (4C)	
	50% N_2_ step	50% N_2_ step	10% N_2_ step
Generalized constrained	0.19 ± 0.15	0.07 ± 0.03	0.08 ± 0.006
Generalized unconstrained	0.55 ± 0.15	0.12 ± 0.01	0.12 ± 0.004
Classical	1.21 ± 0.03	0.16 ± 0.01	0.16 ± 0.003
Classical + inspired fraction	1.20 ± 0.02	0.15 ± 0.02	0.16 ± 0.002

**Fig. 4:**
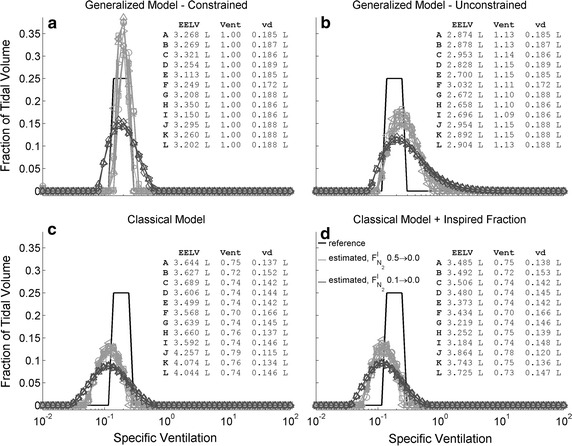
Distribution of specific ventilation estimated from the N_2_ washout of a four compartments physical model. Results from each of twelve repetitions are represented (A to I, in light gray, with $F_{{N_{2} }}^{I}$ step from 0.5 to 0, and J to L, in dark gray, with $F_{{N_{2} }}^{I}$ step from 0.1 to 0), with different symbols for each test. The reference distributions are shown in black. EELV is the end-expiratory lung volume; vent is the sum of the fractional compartmental ventilations (∑γ); and v_d_ is the dead space volume estimated by: Fowler’s technique (for the generalized model, panels **a** and **b**) or the complement of total ventilation (for the classical model, panels **c** and **d**)

For large N_2_ step changes (9 washouts, corresponding to cases A to I), the results for the model 4C were analogous to those for 1C. The constrained generalized model estimated v/V matching the expected specific ventilation, although narrower (Fig. 4a); the unconstrained generalized model underestimated EELV and overestimated the total ventilation by 13%, causing a rightward-shifted and broadened estimated v/V (Fig. 4b). The distribution estimated with the classical model was broader than expected and shifted leftwards from the actual distribution (Fig. 4c, d); the EELV was overestimated by 15% and v_d_ was underestimated by 6%. Again, including the measured inspired N_2_ in the classical model partially corrected EELV estimation reducing the errors to 7%. For N_2_ step changes limited to 10% (3 washouts, Fig. 4, corresponding to the cases J to L), all estimated distributions with the generalized as well as the classical approach resulted broadened (Fig. 4) and with larger sums of squared errors relative to the real distribution (Table 1), indicating the deleterious effect of a decreased signal-to-noise ratio on the estimates. All individual estimated distributions are shown in the Additional file 1: Figures S5–S8.

## Discussion

We proposed a bench comparison between a novel generalized mathematical model for the MBN_2_W [5] and a classical all-parallel model [1]. The tests were performed with a commercial intensive care unit ventilator and physical models mimicking lungs with one or four parallel compartments and a common series dead space. The main results are: (1) the retrieved v/V distribution with the constrained generalized approach was practically coincidental with the actual v/V distribution for both physical models for high N_2_ step changes; the unconstrained solution did not represent the expected distributions, missing the true values of EELV and V_T_; (2) the v/V distribution retrieved with the classical approach was leftward shifted and broader, as compared to the actual, and its corresponding estimates of EELV were slightly favored when the non-ideal step change of N_2_ at the washout onset was taken into account.

We used estimates of respiratory mechanics to provide a first assessment of the reproducibility of the tests and of the assumption of equal ventilation to each of the compartments in 4C. The small spread shows that the physical properties of the models may be considered constant along the washout repetitions, while the fourfold decrease in elastance in 4C compared to 1C suggests that all four anesthetic bags have similar compliances and, consequently, similar ventilations.

The anatomy of the airways consists of a network of ramifications where a strictly common dead space is restricted only to the trachea [7]. The set of subdivisions from the main bronchi to the deeper bronchioles results, during the expiration, in a mixture of alveolar gases originated from their respective airways. Thus, assuming the totality of the anatomical dead space simply as a common series duct is a considerable simplification, even though, as reported by Fortune and Wagner [8], most of the dead space lies proximal to the carina. Nevertheless, the lungs, as represented by the classical model (alveoli connected to the airways opening and the airways as one additional parallel compartment), is less corresponding to the reality. In the present experiments, the physical models agreed very well to the proposed mathematical model, since most of the tubings comprise the common dead space.

Because of the lack of correspondence between the classical model and the actual anatomy, two features arise: the retrieved distribution is shifted to the left as previously reported [8] and broadened as compared to the expected. The specific ventilation of the estimated 1C compartment was close to the theoretically predicted specific ventilation (see Fig. 3c). The spread of the distribution is influenced by factors inherent to the model, such as the difference in sensitivity to the common dead space for slow and fast compartments and the mixing of the contents of the compartments, which decreases the differences between the compartmental washout curves. The distribution curve is also sensitive to choices in data processing, for example the regularization gain used for the estimation of the parameters. The present gains were chosen on the basis of previously simulated experiments [5]. This may be a critical parameter in what concerns the shape of the estimated curve of v/V distribution, particularly its breadth and smoothness. Nevertheless, a tradeoff between accuracy and sensitivity to noise and artifacts is expected, hence this choice should be subjected to further investigations.

The distribution recovering technique applied to the classical model is essentially unconstrained. The solution includes the estimates of EELV and the parallel dead space of the distribution. This dead space does not necessarily correspond to v_d_, representing the ventilation of a compartment with an infinite specific ventilation [3]. Regarding the EELV estimates, they were always overestimated with the classical model. EELV alone has been increasingly regarded as a useful parameter to evaluate the overall lung aeration [9], and it may be straightforwardly calculated by the breath-by-breath summation of the net N_2_ (or other inert gas) volumes expired during the washout.

For the generalized model of MBN_2_W, the EELV that serves as input to the constrained least squares estimation was calculated as above. The EELV estimates resulted accurate for both physical models. Gas exchange calculations based on measurements of gas concentrations and flow rate are very sensitive to the correction of the time delay between these signals [10]. A mainstream capnometer, currently a usual instrument in mechanical ventilation, was used as the time reference to synchronize the mass spectrometer measurements with the flow rate. This time correction, using just the maximal cross-correlation between the CO_2_ concentration signals from the capnometer and the mass spectrometer, revealed feasible and reliable (EELV error < 5% and variability between repetitions < 10% [6]). Alternatively, an ultrasound flowmeter monitoring the washout of sulfur hexafluoride (SF_6_), an inert and insoluble gas with a high molecular mass compared to the ambient air components, may be used. This device allows simultaneous and synchronous measures of flow rate and SF_6_ concentration and has been used for the estimation of ventilatory inhomogeneity [11, 12].

Breath-by-breath estimates of the series dead space is a requirement for both the constrained and the unconstrained generalized v/V distribution. Instead of using prediction formulae, a direct measurement of that dead space is recommended, for example by applying Fowler’s technique [4] to the capnogram [5] as in the present work. Prediction formulae are scarce and inaccurate, especially for some conditions such as during mechanical ventilation, in which body position varies and EELV depends on the applied PEEP. For instance, there are conflicting reports as to the effect of the dead space on a vastly employed index to quantify ventilatory inhomogeneity, the lung clearance index (LCI). Despite Haidopoulou et al. [11] concluded that LCI is minimally affected by airway dead space, Neumann et al. [12] found an association between LCI and v_d_/V_T_. The LCI is an overall index of ventilatory inhomogeneity; in theory the increase of v_d_/V_T_ should increase the magnitude of LCI. As an alternative, the alveolar lung clearance index (aLCI) [11] was proposed by considering the alveolar ventilation instead of the total ventilation as the bulk flow washing the alveolar units. The present generalized approach is based on the same assumption. Notably, an error in v_d_ estimation will result in a shifted distribution [5], as demonstrated with the extreme case of the classical model. Likewise, if v_d_ is overestimated the shift will be to the right (Additional file 1: Figure S9) due to slower washout (increased rebreathing) for each modeled v/V.

The v/V distribution of the respiratory system may be modeled by a continuous curve within a finite interval. The recovery of this distribution from the limited information present in a MBN_2_W is an ill-posed problem and requires simplifying assumptions. The three assumptions relevant to the estimation method are smoothness, known bounds and discrete representativity. The first assumption was discussed above. The bounds used here are the same from [1, 13], and clearly will lead to wrong estimates if they don’t encompass all the v/V ratios of the real compartments. The a priori choice of 50 compartments is usual in the literature [1, 3, 13, 14]. In this study we tried to match every physical compartment to values present in the chosen 50 v/V ratios, favoring estimation: mismatch(es) between the physical v/V ratios and the set of chosen v/V ratios in the mathematical model will, in general, cause the true ratio to be represented by a combination of modeled compartments. This should affect mainly the amplitude and breadth of the distribution, and less its location. An example of the effects of such mismatch can be seen in Additional file 1: Figure S10.

Some limitations are addressed hereupon. To our best knowledge, this is the first report on multiple-breath washout of a multicompartmental physical model. Hence, we could not discuss our results against the literature as to possible comparative improvements. The physical models were limited to up to 4 units and this is far from the number of units found in experimental works with humans [3, 14–16]. Considering that the v/V is distributed on a log scale, a simulation with many more units would be difficult to perform in view of the present method of construction of v/V units. For the estimation of v/V distribution we used the same cycles selected for calculating the EELV [6]. For our combination of V_T_ and compartments’ volumes, this choice lead to a larger number of cycles than the commonly used of 17 [1, 2], which could have favored our results. Numerical simulations showed that, for the generalized model, both choices of cycles have similar estimations, although 17 cycles respected more the number of modes [5]. In the Additional file 1: Figures S1–S8, we show that this equivalence holds true in our experimental condition, including for the classical model. Lastly, one of the features of the generalized approach to estimate v/V distributions is that V_T_ and EELV are not necessarily constrained to be constant, as in the classical method. The present results did not include tests with variable ventilation [17] feasible at the laboratory since commercial mechanical ventilators currently feature this choice of strategy.

## Conclusions

In conclusion, the present work compared the v/V distributions estimated by both the classical and generalized approaches employing experimental data obtained with in vitro models. The method that resulted in better coincidence with the actual distribution was the generalized approach with a constrained least squares solver with imposed EELV and V_T_.

## Additional file

**Additional file 1.** The individual estimates of specific ventilation distributions are shown for each combination of physical and mathematical model, also considering estimates with 17 breath cycles. All estimates of end-expiratory lung volume, total ventilation and dead space are tabulated, together with the reference values. Sensitivity to error in estimated v_d_ and to the number N of modeled compartments.

## Abbreviations

MBN_2_W: multiple-breath nitrogen washout
EELV: end-expiratory lung volume
v_d_: series dead space
v/V: ventilation-to-volume
V_T_: tidal volume
N: number of modeled alveolar units
Vol: alveolar unit end-expiratory volume
*γ*: alveolar unit fraction of tidal volume
J: index of alveolar unit
*S*: specific ventilation
*k*: index of breath cycle
$F_{{N_{2} }}^{A}$: alveolar unit concentration of N_2_
$F_{{N_{2} }}^{et}$: end-tidal N_2_ concentration
$F_{{N_{2} }}^{I,A}$: alveolar unit inspired N_2_ concentration
$F_{{N_{2} }}^{I}$: ventilator delivered N_2_ concentration
