# Supplementary material for: Generalized estimation of the ventilatory distribution from the multiple-breath washout: a bench evaluation study
Source: Biomed Eng Online. 2018 Jan 15;17:3. doi: 10.1186/s12938-018-0442-3 (PMC5769492; doi:10.1186/s12938-018-0442-3)
Supplement: Supplementary file 1 — Additional file 1. The individual estimates of specific ventilation distributions are shown for each combination of physical and mathematical model, also considering estimates with 17 breath cycles. All estimates of end-expiratory lung volume, total ventilation and dead space are tabulated, together with the reference values. Sensitivity to error in estimated vd and to the number N of modeled compartments. [file 12938_2018_442_MOESM1_ESM.pdf]

.

Supplementary Online Material

Generalized estimation of the ventilatory distribution from the multiple- breath washout: a bench evaluation study

Gabriel Casulari Motta- Ribeiro, Frederico Caetano Jandre, Hermann Wrigge and Antonio Giannella-Neto

1. Individual results for the single-compartment model (1C)

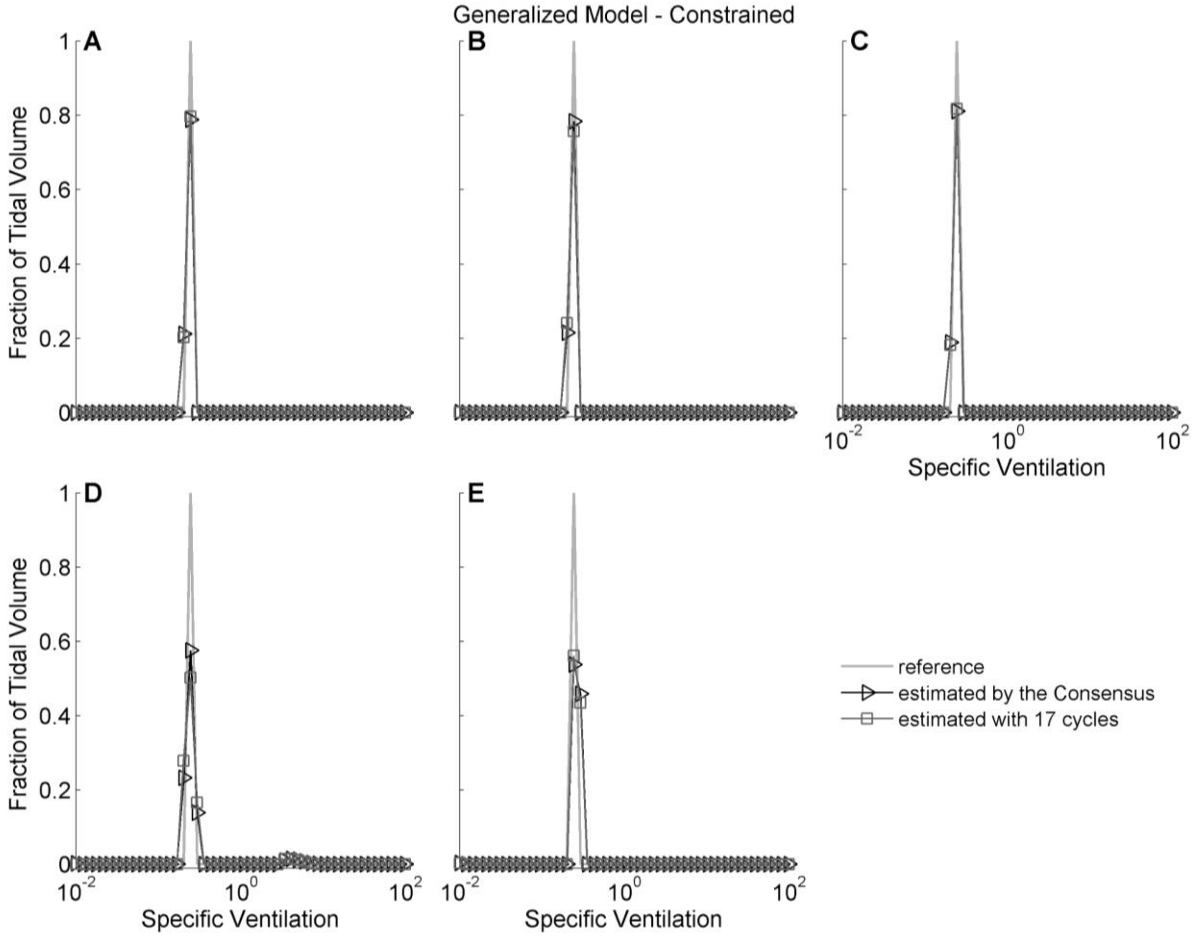

Figure S1 - Distribution of specific ventilation estimated from each of five N_2 washouts of a single-compartment physical model using the constrained generalized mathematical model with 50 compartments. The distributions were estimated using either the number of cycles according to the Consensus [1], that is, until 1/40th of the initial N_2 concentration (black triangles) or alternatively 17 cycles (gray squares). The reference distribution is shown in light gray.

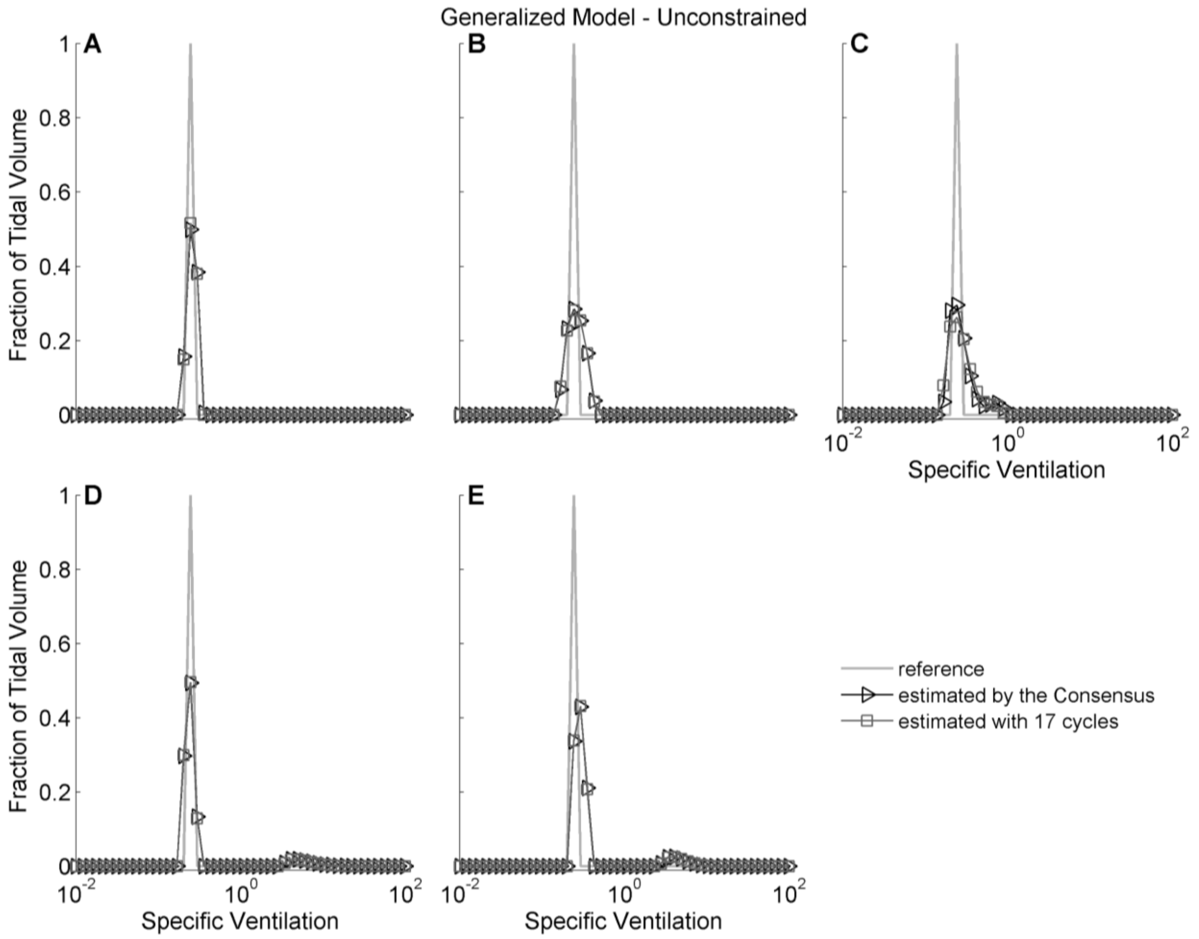

Figure S2 - Distribution of specific ventilation estimated from each of five N_2 washouts of a single-compartment physical model using the unconstrained generalized mathematical model with 50 compartments. The distributions were estimated using either the number of cycles according to the Consensus [1], that is, until 1/40th of the initial N_2 concentration (black triangles) or alternatively 17 cycles (gray squares). The reference distribution is shown in light gray.

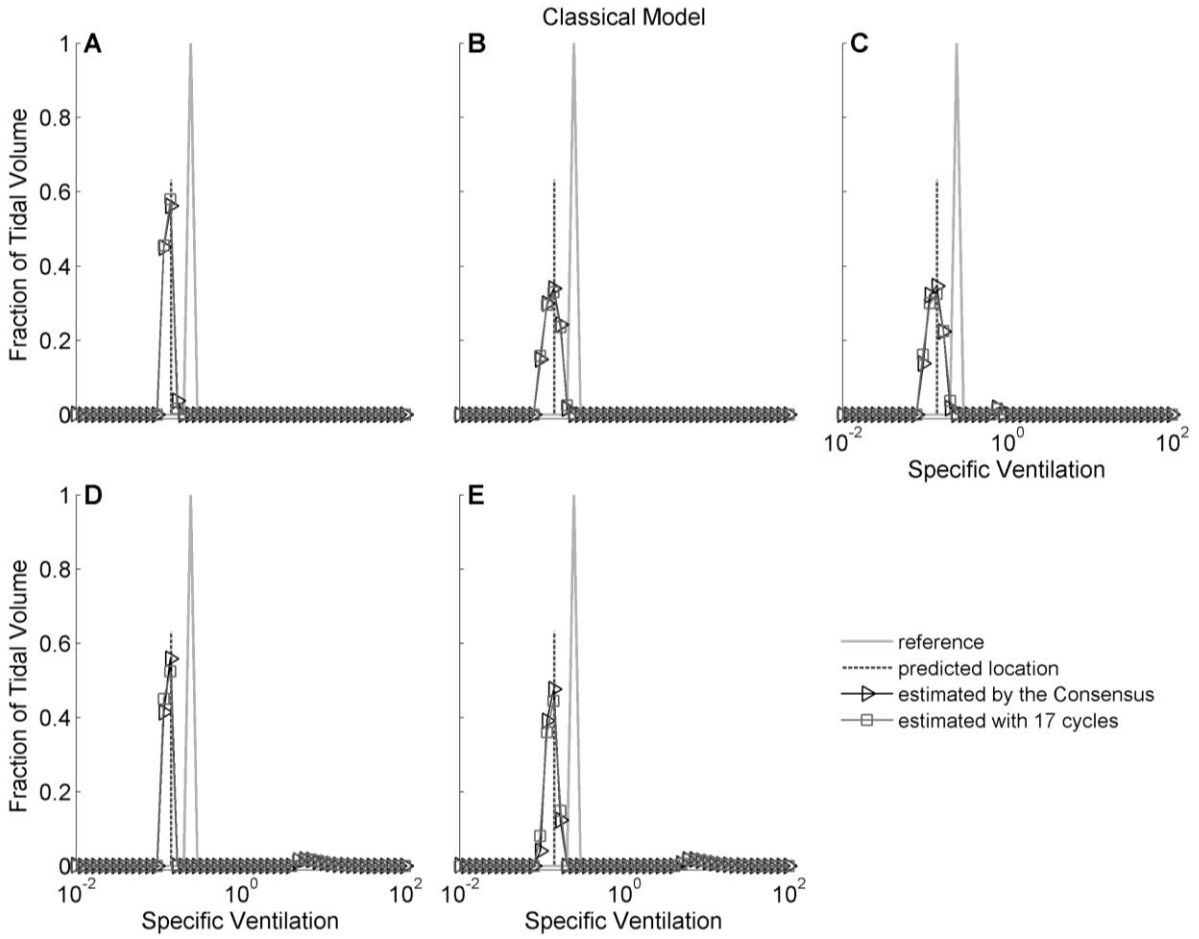

Figure S3 - Distribution of specific ventilation estimated from each of five N_2 washouts of a single-compartment physical model using the classical all parallel mathematical model with 50 compartments. The distributions were estimated using either the number of cycles according to the Consensus [1], that is, until 1/40th of the initial N_2 concentration (black triangles) or alternatively 17 cycles (gray squares). The reference distribution is shown in light gray. The dashed vertical line represents the theoretical prediction for the compartment estimated with the classical model if there is a series dead space in the actual system.

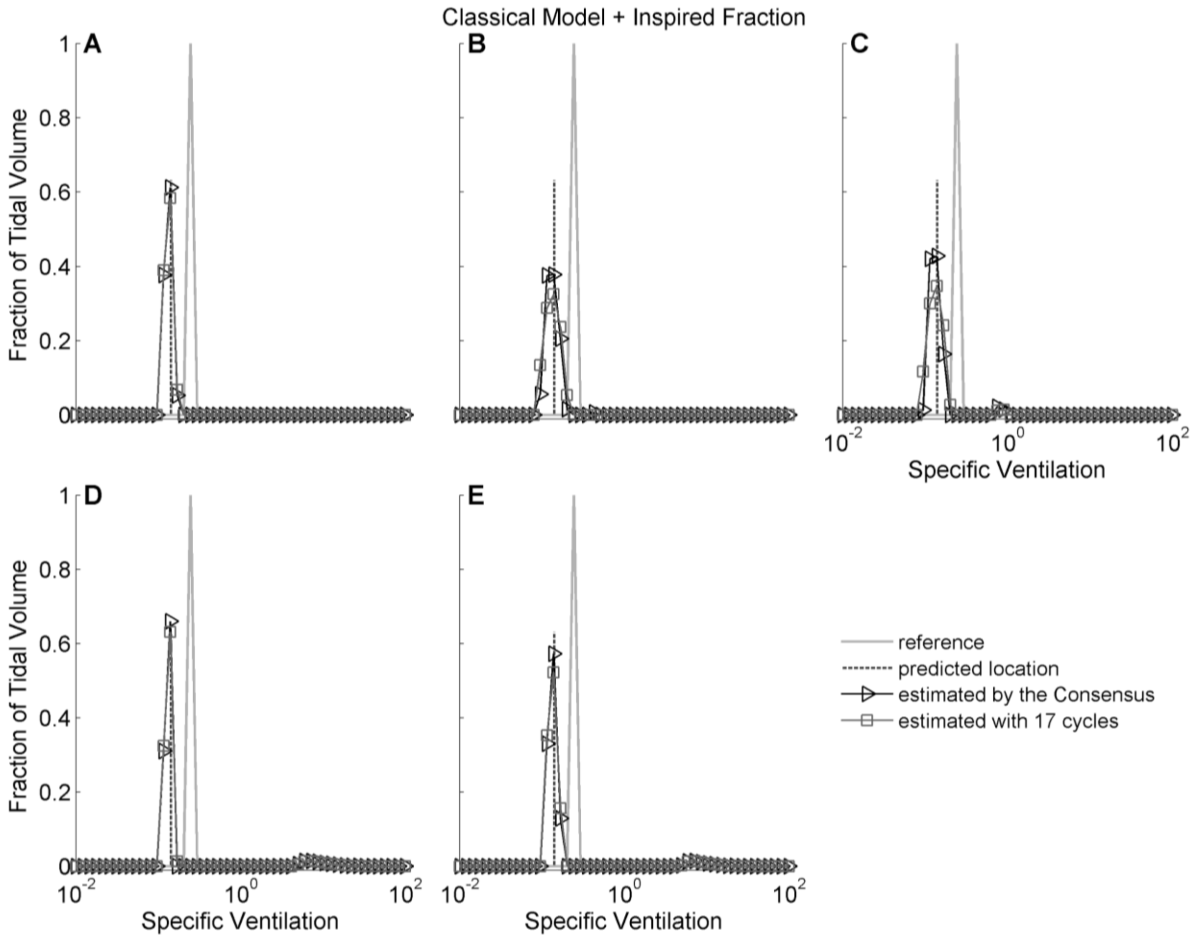

Figure S4 - Distribution of specific ventilation estimated from each of five N_2 washouts of a single-compartment physical model using the classical all parallel mathematical model with 50 compartments, and considering the measurements of the inspired N_2 concentration. The distributions were estimated using either the number of cycles according to the Consensus [1], that is, until 1/40th of the initial N_2 concentration (black triangles) or alternatively 17 cycles (gray squares). The reference distribution is shown in light gray. The dashed vertical line represents the theoretical prediction for the compartment estimated with the classical model if there is a series dead space in the actual system.

Table S1 - End expiratory volume, total ventilation and dead space for each washout model in all five experiments with the single-compartment physical model. End expiratory volume is the sum of the estimated compartmental volumes; total ventilation is the sum of the estimated compartmental fractions of ventilation (unitary, in the constrained case); and dead space is estimated from the capnograms using Fowler's method (constrained and unconstrained cases) or from the complement to the total ventilation and the measured tidal volume (classical and classical + inspired fraction cases). Reference values are the expected from the geometry of the physical model.

		Experiments					Reference
		A	B	C	D	E	
End expiratory volume (L)	Constrained	1.125	1.156	1.119	1.140	1.133	1.092
	Unconstrained	1.093	1.091	1.07	1.077	0.96	
	Classical	1.353	1.408	1.414	1.383	1.226	
	Classical + inspired fraction	1.314	1.322	1.333	1.291	1.182	
Total ventilation	Constrained	1.00	1.00	1.00	1.00	1.00	1.00
	Unconstrained	1.05	1.04	1.07	1.02	1.08	
	Classical	0.68	0.72	0.69	0.65	0.60	
	Classical + inspired fraction	0.67	0.72	0.68	0.65	0.60	
Dead space (mL)	Constrained	71	70	69	73	85	92
	Unconstrained	71	70	69	73	85	
	Classical	79	70	76	89	100	
	Classical + inspired fraction	80	70	78	89	101	

2. Individual results for the four-compartments model (4C)

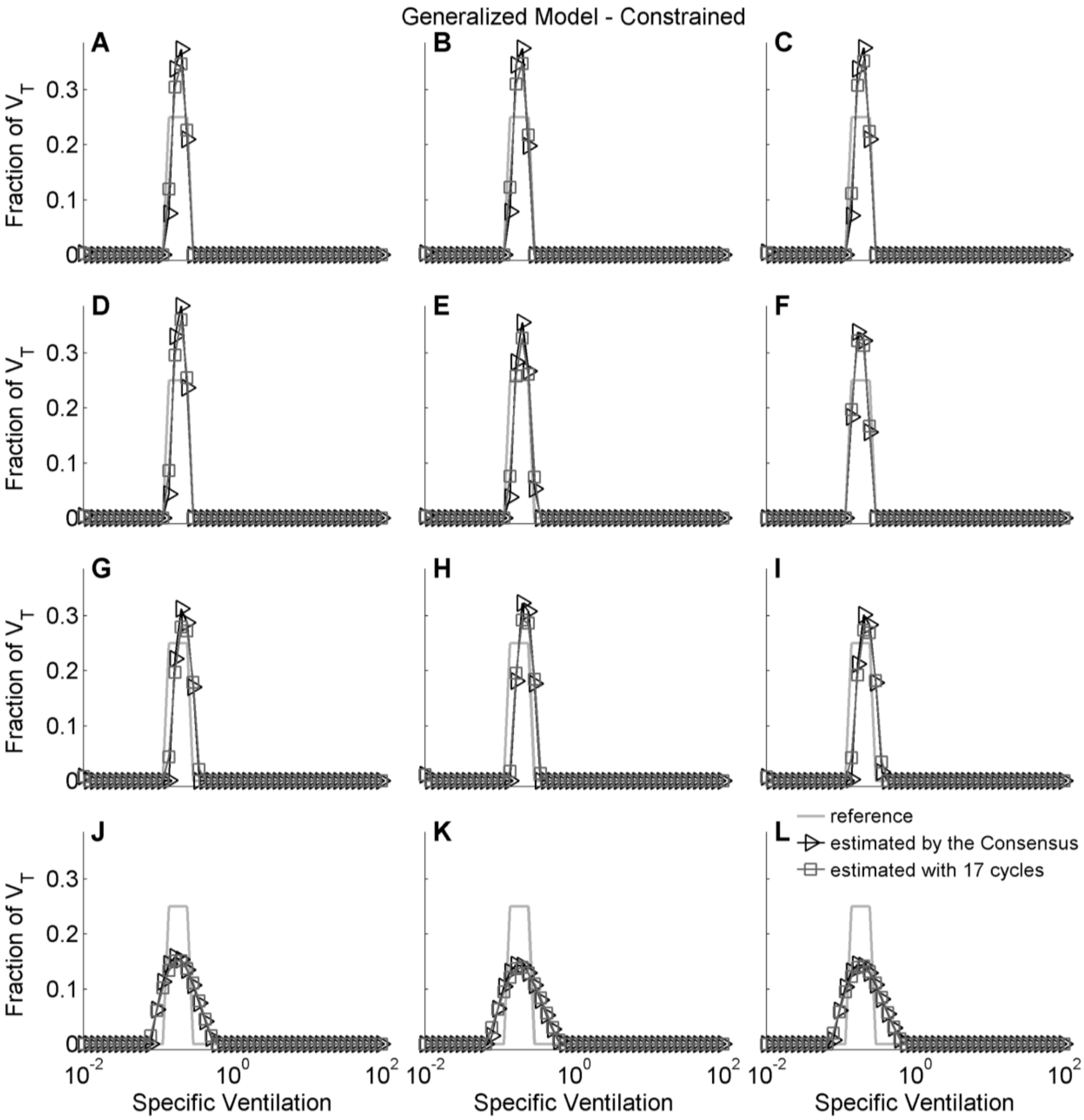

Figure S5 - Distribution of specific ventilation estimated from each of twelve N_2 washouts of the four-compartment physical model using the constrained generalized mathematical model with 50 compartments. The distributions were estimated using either the number of cycles according to the Consensus [1], that is, until 1/40th of the initial N_2 concentration (black triangles) or alternatively 17 cycles (gray squares). The reference distribution is shown in light gray.

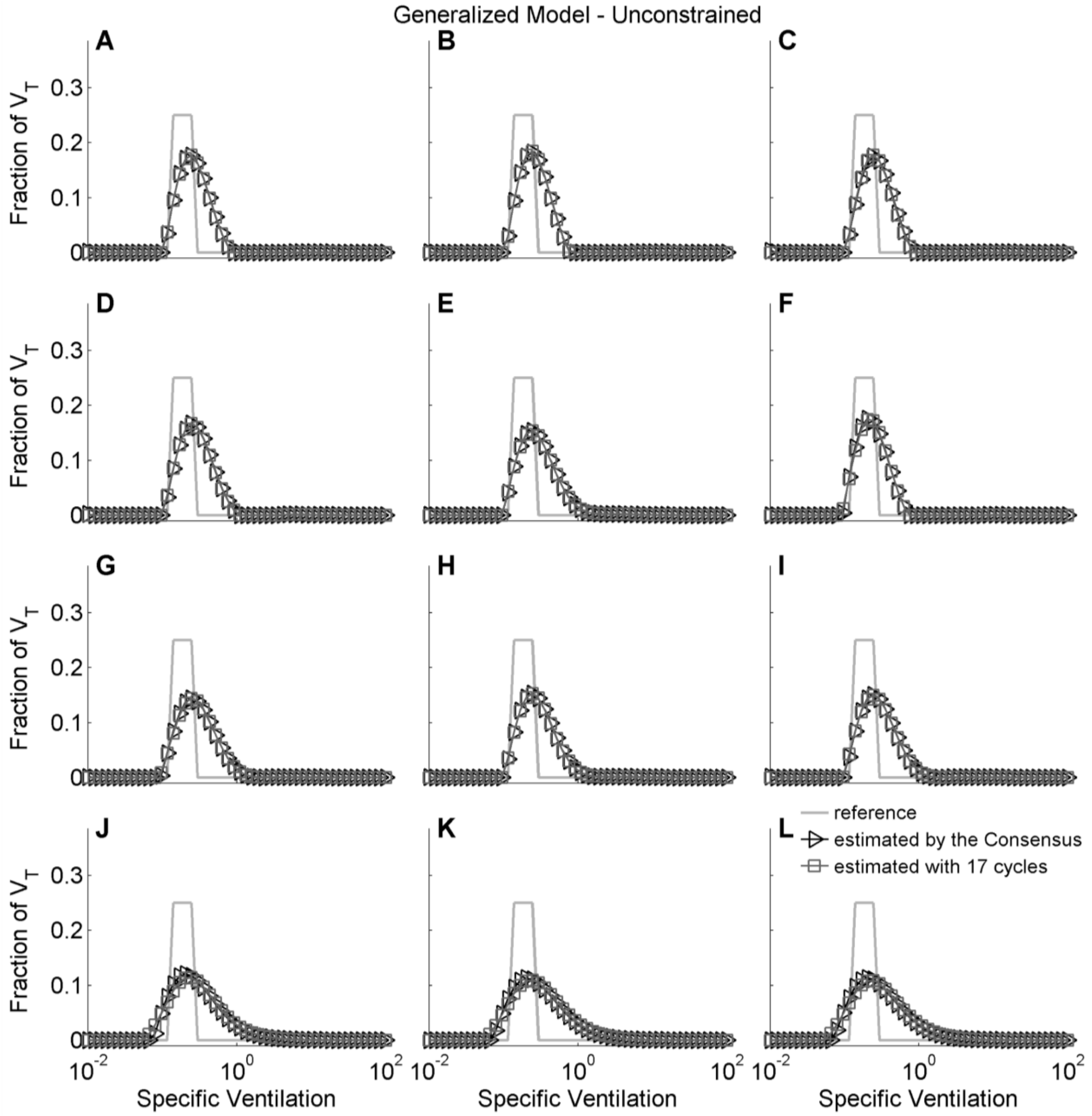

Figure S6 - Distribution of specific ventilation estimated from each of twelve N_2 washouts of the four-compartment physical model using the unconstrained generalized mathematical model with 50 compartments. The distributions were estimated using either the number of cycles according to the Consensus [1], that is, until 1/40th of the initial N_2 concentration (black triangles) or alternatively 17 cycles (gray squares). The reference distribution is shown in light gray.

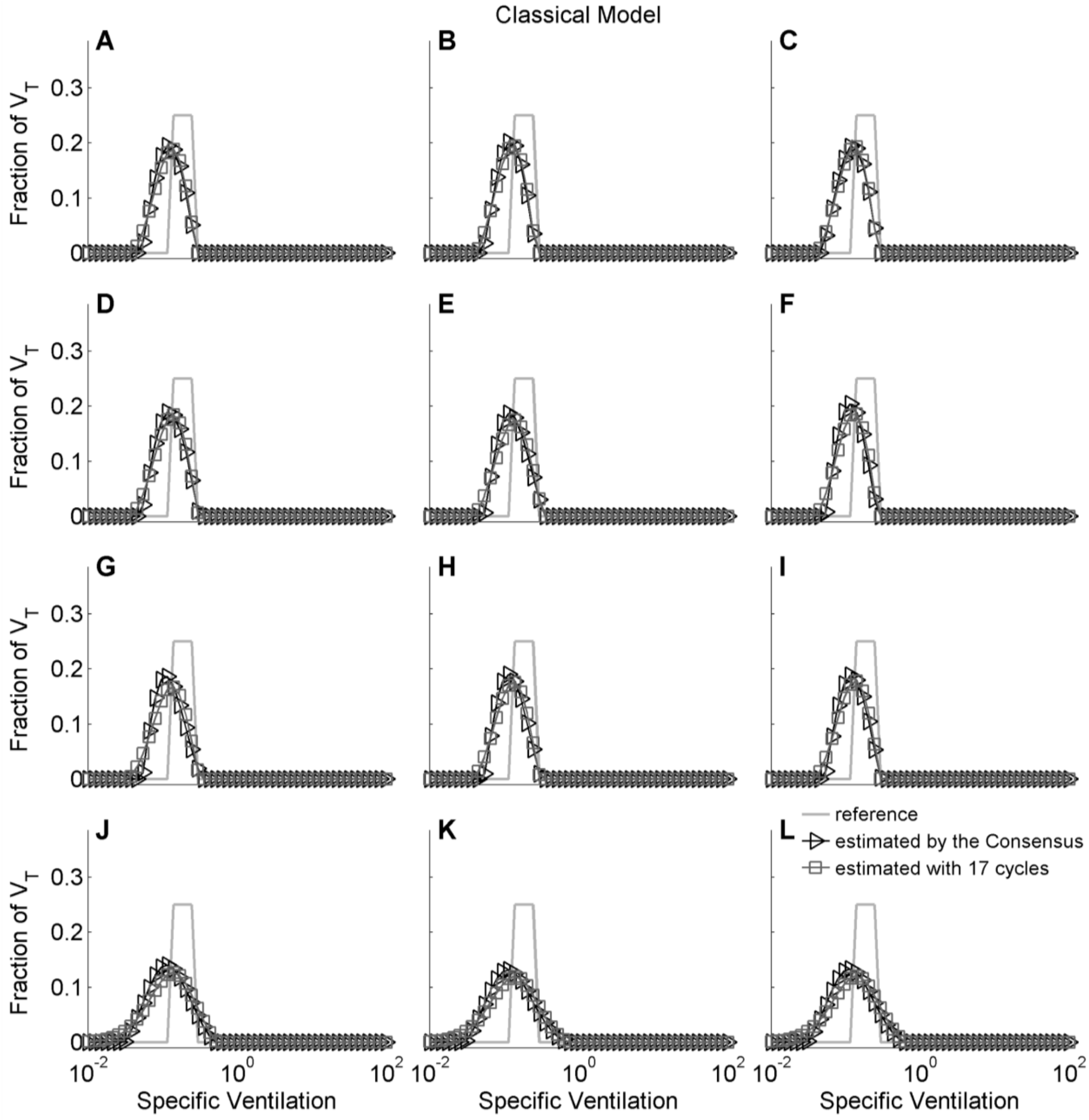

Figure S7 - Distribution of specific ventilation estimated from each of twelve N_2 washouts of the four-compartment physical model using the classical all-parallel mathematical model with 50 compartments. The distributions were estimated using either the number of cycles according to the Consensus [1], that is, until 1/40th of the initial N_2 concentration (black triangles) or alternatively 17 cycles (gray squares). The reference distribution is shown in light gray.

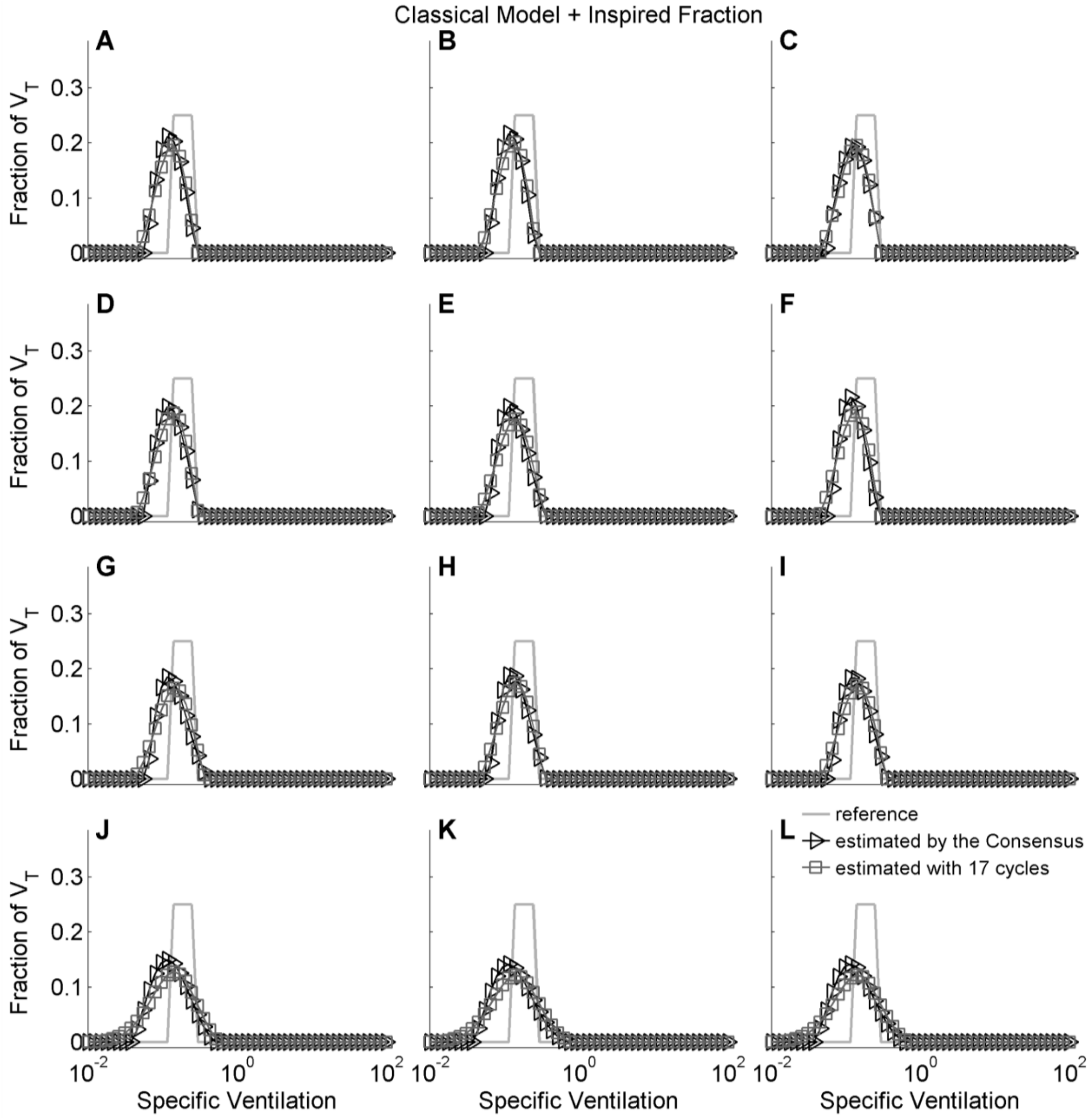

Figure S8 - Distribution of specific ventilation estimated from each of twelve N_2 washouts of the four-compartment physical model using the classical all-parallel mathematical model with 50 compartments, accounting for the measured inspired concentration of N_2. The distributions were estimated using either the number of cycles according to the Consensus [1], that is, until 1/40th of the initial N_2 concentration (black triangles) or alternatively 17 cycles (gray squares). The reference distribution is shown in light gray.

Table S2 - End expiratory volume, total ventilation and dead space for each washout model in all twelve experiments with the four-compartment physical model. End expiratory volume is the sum of the estimated compartmental volumes; total ventilation is the sum of the estimated compartmental fraction of ventilation (unitary, in the constrained case); and dead space is estimated from the capnograms using Fowler's method (constrained and unconstrained cases) or from the complement to the total ventilation and the measured tidal volume (classical and classical + inspired fraction cases). Reference values are the expected from the geometry of the physical model.

		Experiments												Reference
		A	B	C	D	E	F	G	H	I	J	K	L	
End expiratory volume (L)	Constrained	3.268	3.269	3.321	3.254	3.113	3.249	3.208	3.350	3.150	3.295	3.260	3.202	3.242
	Unconstrained	2.874	2.878	2.953	2.828	2.700	3.032	2.672	2.658	2.696	2.954	2.892	2.904	
	Classical	3.644	3.627	3.689	3.606	3.499	3.568	3.639	3.660	3.592	4.257	4.074	4.044	
	Classical + inspired fraction	3.485	3.492	3.506	3.480	3.373	3.434	3.219	3.252	3.184	3.864	3.743	3.725	
Total ventilation	Constrained	1.00	1.00	1.00	1.00	1.00	1.00	1.00	1.00	1.00	1.00	1.00	1.00	1.00
	Unconstrained	1.13	1.13	1.14	1.15	1.15	1.11	1.10	1.10	1.09	1.15	1.15	1.13	
	Classical	0.75	0.72	0.74	0.74	0.74	0.70	0.74	0.76	0.74	0.79	0.76	0.74	
	Classical + inspired fraction	0.75	0.72	0.74	0.74	0.74	0.70	0.74	0.75	0.74	0.78	0.75	0.73	
Dead space (mL)	Constrained	185	187	186	189	185	172	188	186	186	188	188	188	152
	Unconstrained	185	187	186	189	185	172	188	186	186	188	188	188	
	Classical	137	152	142	144	142	166	145	137	146	115	134	146	
	Classical + inspired fraction	138	153	142	145	142	166	146	139	148	120	136	147	

3. Sensitivity to error in estimated series dead space (v_d)

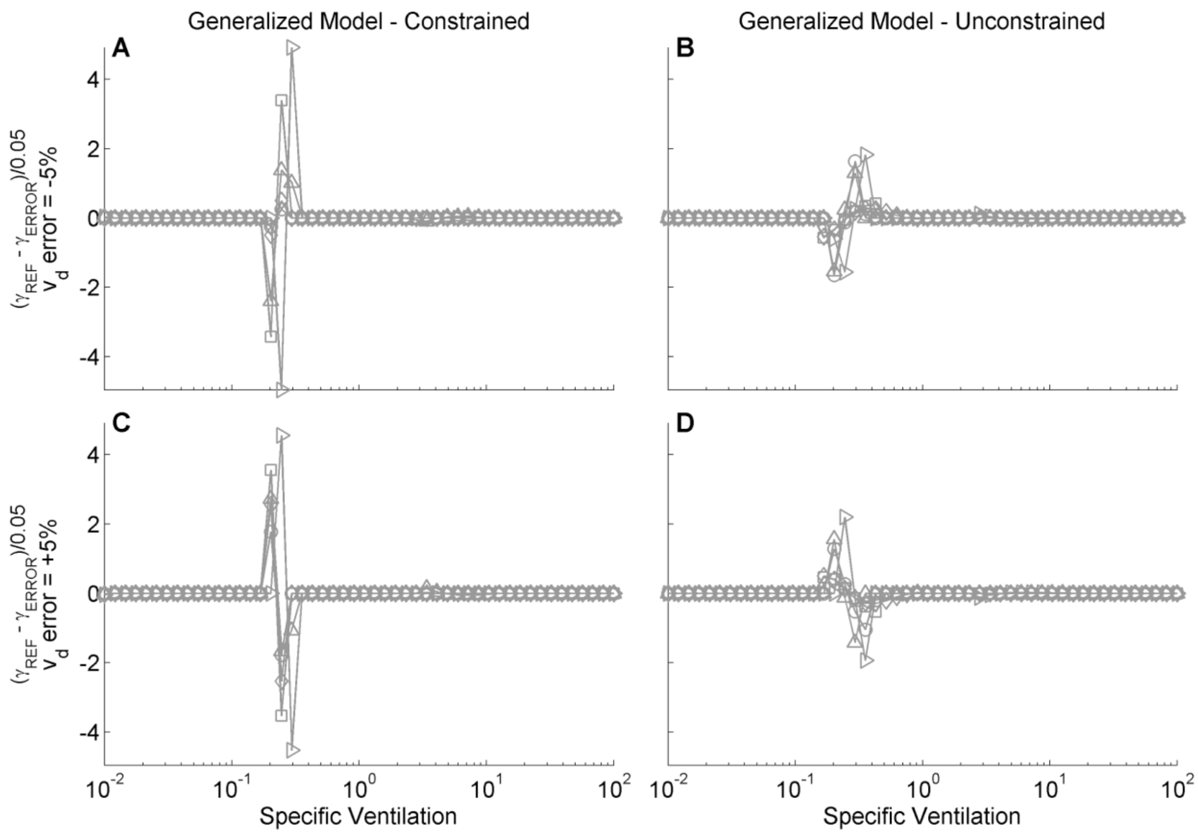

Figure S9 - Sensitivity of estimated specific ventilation distribution to the error in series dead space estimation. The graphs show the difference between the fractions of tidal volume estimated with the measured v_d (γ_{REF}, distributions shown in the main paper and in Figures S1-S2) and those estimated with -5% or +5% deviation in v_d (γ_{ERROR}) (respectively, A and B, and C and D), divided by 5%. For both constrained and unconstrained estimates, an overestimation in v_d shifts the distribution toward fast compartments (right side), and vice-versa.

4. Sensitivity to the number of modeled compartments (N) for 1C and 4C

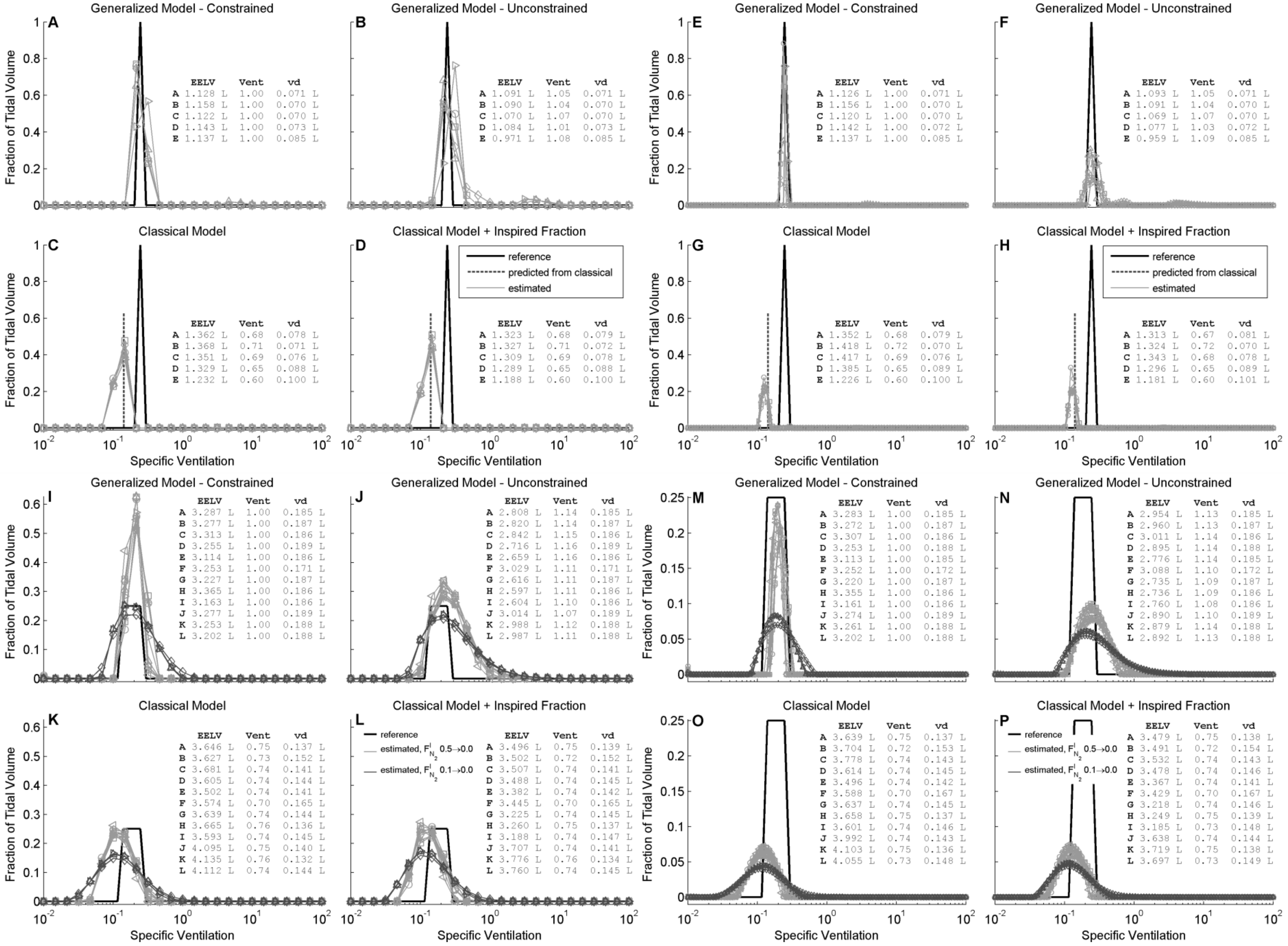

Figure S10 - Distribution of v/V estimated from N_2 washouts of a single- (upper panels) or four- (lower panels) compartment physical model. The distributions were estimated using the classical all-parallel model or the novel generalized model with series v_d. The constrained version of the last model imposes a solution with the measured v_d and EELV, and a unitary total ventilation (Vent). Reconstruction with 25 (panels A,B,C,D,I,J,K,L) or 100 (panels E,F,G,H,M,N,O,P) compartments. For the four-compartment model, inspired N_2 (F^I_{N2}) varied by 50% (light gray) or 10% (dark gray). In all cases, the number of cycles used for estimation was determined according to the Consensus [1], that is, until 1/40th of the initial N_2 concentration. The reference distribution, expected from the geometries of the physical models, is shown in black.

References

1. Robinson PD, Latzin P, Verbanck S, Hall GL, Horsley A, Gappa M, et al. Consensus statement for inert gas washout measurement using multiple- and single- breath tests. Eur Respir J. 2013;41:507–22. DOI:10.1183/09031936.00069712.
